# Research Achievements of Oral Submucous Fibrosis: Progress and Prospect

**DOI:** 10.1155/2021/6631856

**Published:** 2021-03-18

**Authors:** Hui Xu, Feng-yuan Lyu, Jiang-yuan Song, Yu-ming Xu, Er-hui Jiang, Zheng-Jun Shang, Li-li Chen, Zhi Xu

**Affiliations:** ^1^Department of Stomatology, Union Hospital, Tongji Medical College, Huazhong University of Science and Technology, Wuhan 430022, China; ^2^School of Stomatology, Tongji Medical College, Huazhong University of Science and Technology, Wuhan 430030, China; ^3^Hubei Province Key Laboratory of Oral and Maxillofacial Development and Regeneration, Wuhan 430022, China; ^4^Center of Stomatology, Tongji Hospital, Tongji Medical College, Huazhong University of Science and Technology, Wuhan 430030, China; ^5^The State Key Laboratory Breeding Base of Basic Science of Stomatology (Hubei-MOST) & Key Laboratory for Oral Biomedicine Ministry of Education, Wuhan University, Wuhan, China

## Abstract

Oral submucous fibrosis (OSMF) is a kind of chronic, insidious disease, and it is categorized into potentially malignant disorders (PMD), which poses a global and regional problem to public health. It is considered to be a multifactorial disease, such as due to areca nut chewing, trace element disorders, and genetic susceptibility. However, there is still no unanimous conclusion on its pathogenesis, diagnosis, and treatment strategies. Hence, this article provides a comprehensive review and prospect of OSMF research, providing scholars and clinicians with a better perspective and new ideas for the research and treatment of OSMF.

## 1. Introduction

Oral submucous fibrosis (OSMF) is a chronic and latent malignant disease, which poses a global and regional problem to public health, especially in East and Southeast Asia where areca nut chewing is popular. The malignant transformation rate of OSMF to oral squamous cell carcinoma (OSCC) accounts for 7%-13% [1].

Fibrosis and hyalinization of subepithelial tissue are the most crucial clinicopathological features of OSMF, which greatly affect the patients' quality of life. The appearance of myofibroblasts and the consistent expression of *α*-smooth muscle actin (*α*-SMA) are considered to be signs of progressive fibrosis and are thought to cause the change of OSMF microenvironment, leading to tumorigenesis [2]. Even though the exact etiology and pathogenesis of OSMF have not yet been elucidated totally, the currently recognized pathogenic factor is areca nut. Meanwhile, great progress of OSMF-relevant diagnosis and clinical treatment has been reported in recent years.

Comprehensive understanding of OSMF research progress not only provides clinicians with a better horizon but also provides new insights into the OSMF researches and treatment. This article provides a comprehensive review and prospect of the future researches of OSMF based on the latest vision of relative medical progress.

## 2. Etiopathogenesis

OSMF is the first demonstrated oral disease which is related with sustained effect by the irritants on the oral mucosa. Numerous studies that clarified its etiopathogenesis have focused on the extracellular matrix (ECM) with increased synthesis of subepithelial collagen and decreased collagen degradation. Though the etiopathogenesis of OSMF is still not completely clear, the current general view is that OSMF development is the result of a combination of factors, including chewing areca nut, copper toxicity, vitamin deficiency and malnutrition, anemia, and genetic susceptibility.

### 2.1. Lifestyle

Numerous epidemiological studies have provided evidence that areca nut is the main causative agent of OSMF. Areca nut contains a variety of alkaloids, of which arecoline content is the most abundant; a large number of studies have shown that arecoline plays an important role in the etiopathogenesis of OSMF [3–5]. It is estimated that hundreds of millions of people worldwide use areca nut products [6]. In China, almost all OSMF patients have the habit of chewing areca nut [6], and most of the patients are male, while the most common lesion site is the buccal mucosa [7]. There is a clear dose-dependent relationship between OSMF incidence rate and the frequency of chewing areca nut [8].

Studies have also found that chewing dried areca nuts are more pathogenic and carcinogenic than chewing fresh areca nuts, and the extremely harmful ingredients in areca nuts play a vital role in the occurrence of oral mucosal diseases and oral cancer [9]. In *in vitro* experiments, it has been proven that areca nut extract (ANE) induces fibrosis marker gene *α*-smooth muscle actin (*α*-SMA) and connective tissue growth factor (CTGF) expression [10]. In *in vivo* experiments, subcutaneous injection of areca nut extract (ANE) successfully induced skin fibrosis, showing similar characteristics to OSMF [11].

A myriad of studies has shown that arecoline, as the most abundant alkaloid in areca nut, plays an important role in the pathogenesis of OSMF. The role of arecoline in the pathogenesis of OSMF is mainly through the activation of fibroblasts and the increase of collagen. The key cells in the OSMF microenvironment are recognized as activated fibroblasts, which were called myofibroblasts, with a highly contracted phenotype, characterized by the presence of developed microfibrils and *α*-SMA [12]. According to recent studies, arecoline has been proven to induce buccal mucosa fibroblast activation [13]. Simultaneously, it can also increase the tissue inhibitors of matrix metalloproteinase-1 (TIMP-1) which reduces collagen degradation and induces extracellular matrix (ECM) deposition [12]. In addition, it has antiproliferative and cytotoxic effects on endothelial cells, which may lead to impaired vascular function, thus participating in the pathogenesis of OSMF [3].

**Figure 1:**
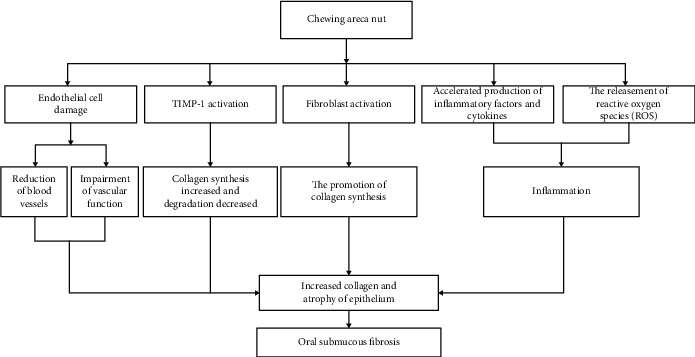
The etiopathogenesis of OSMF caused by chewing areca nut.

Studies have also shown that inflammatory factors participate in the initiation process of OSMF. Oral mucosal inflammation caused by chewing areca nut is another key event in the etiopathogenesis of OSMF. So far, it has been observed as primary inflammatory reactions (mucositis) in OSMF patients [14]. The etiopathogenesis of OSMF caused by chewing areca nut is summarized in Figure 1.

#### 2.1.1. Disorder of Trace Elements

It should be noticed that the level of trace elements in the patients with OSMF has changed. It has been found that malnutrition (such as protein and vitamin deficiencies or anemia), specifically changes in serum iron, zinc, and copper, is related to the development of OSMF [15–17]. The reduction of serum iron can change the epithelial structure, increasing mucosal permeability and inhibiting its barrier protection. In OSMF patients, it was demonstrated that iron and serum ferritin levels decrease and total iron binding capacity (TIBC) increases, which is accorded with the clinical stage and histological grade of OSMF [18]. Current research has also found that zinc has the ability to resist fibrosis and can induce the activation of superoxide dismutase (SOD), thereby inhibiting the production of reactive oxygen species (ROS) [19]. The reduction of zinc can reduce the activity of the oral mucosa peroxidase system and inhibit the elimination of certain harmful substances under continuous external ANE stimulation; the oral mucosa is prone to deterioration and fibrosis. Excessive copper was found in tissues of other fibrotic diseases (such as Wilson's disease, child cirrhosis, and primary biliary cirrhosis) [20]. It was considered that copper is a cofactor of lysyl oxidase and promotes collagen synthesis, indicating the relationship between copper and OSMF development [21]. Adding different concentrations of copper *in vitro* has been shown to increase the proliferation ability of fibroblasts [20]. Study using human skin fibroblasts showed that fibroblasts have an effective system for absorbing copper [22]. Intracellular copper may form tissue complexes and then promote upregulation of collagen synthesis and subsequent crosslinking through the lysyl oxidase pathway [22]. A large case-control study confirmed that the incidence of OSMF is positively correlated with the concentration of copper in drinking water [23]. Exposure to a subtle high-normal copper diet such as copper drinking water increases the concentration of copper in local tissues, making oral mucosa prone to OSMF development. It has also been proposed that once the OSMF condition is established, the ability to clear copper is injured [20]. Furthermore, the levels of immunoglobulins G and A in the serum and saliva of OSMF patients increased, while the levels of hemoglobin (HB) and total serum protein (TSP) decreased significantly [24].

#### 2.1.2. Genetic Susceptibility

Studies have shown that OSMF easily occur in people who chew areca nuts, but only some people using areca nuts are susceptible to OSMF [25]. It should be noticed that the symptoms of OSMF disease do not alleviate, even when some OSMF patients stop chewing areca nuts. There is obvious genetic susceptibility in OSMF disease development. OSMF pathogenesis is related to a variety of chromosomal, genetic, and molecular changes [4, 25]. In OSMF tissue samples, cytological changes often occur, especially micronuclei and other nuclear abnormalities, such as binuclear, nuclear fragmentation, and nuclear dissolution [26]. It was found that the frequency of *HLA-A10*, *HLA-B7*, and *HLA-DR3* dominance increased in OSMF patients [27]. Moreover, gene polymorphisms of *COL1A1*, *COL1A2*, *COLase*, *LY oxidase*, *TGF-β1*, and *cystatin C* are associated with OSMF [3, 28].

### 2.2. Prospects of OSMF Etiopathogenesis Researches

Besides the main causes discussed above, other etiological factors include psychological factors, immune factors, frequency and duration of smoking/alcohol, and so on [29]. Though the molecular and cellular mechanisms of OSMF were studied extensively, some aspects still have not been noticed yet. Hence, several directions for future OSMF etiopathogenesis researches are prospected.

At present, the research on the etiopathogenesis of OSMF mainly focuses on the people in the same country or area. The use methods of areca nut have obvious regional characteristics. Is there a difference in the susceptibility of OSMF among people in different regions/races? Is there any difference in OSMF-related oral cancer risk among different populations? Related research needs further clarification. The effects of the areca nut/betel quid ingredients and substitutes on OSMF development and progress have also been proven. However, we should notice that different countries/regions produce different types of areca nut products with different kinds of ways for chewing. The various producing processes and using habits seem to have different effects on OSMF development. The relative studies are limited, especially multiple region-united epidemiology studies. We should warn people of the danger of areca nut usage; on the other hand, we still need to explore relatively safe ways to make use of this herbal drug. Modified production processes of betel quid may have positive impact on global OSMF prevention. The lack of regional and international cooperation limits this kind of researches actually. Also, commercial reason is a factor that cannot be ignored to prompt the issues.

In view of the areca nut's popularity, the high incidence of OSMF will bring huge medical and health pressures to the world, so we need to strive for epidemiological and etiological research with international and regional cooperation, in order to fight against this global health problem.

## 3. Diagnosis

The diagnosis of OSMF is mainly based on subjective clinical diagnosis, while histopathological examination is still the gold standard [30]. OSMF has different clinical and histopathological manifestations, and we can classify and diagnose OSMF through clinical and histopathological characteristics. Clinical symptoms of OSMF include ulcer, dry mouth, burning sensation, and mouth opening limitation [1]. Oral mucosa whitening is an important clinical feature in the early stage of OSMF. In the late stage, mouth opening limitation is caused by fibrosis of oral mucosa area. Pathological features include chronic inflammation, excessive collagen deposition in the submucosal connective tissue of the oral mucosa, local inflammation in the lamina propria or deep connective tissue, and degenerative changes in muscle [31].

### 3.1. OSMF Staging/Classification

In order to accurate clinical classification of OSMF patients to improve the accuracy of diagnosis and individualized treatment, scholars have classified OSMF into stages to differentiate clinical manifestations and pathological manifestations of patients. At present, the clinical, functional and histological stage/classification of OSMF has been recorded and used for the clinical diagnosis and treatment of OSMF [27]. Khanna and Andrade [32] classify OSMF according to maximum interincisal opening (MIO). Pindborg and Sirsat [33] classify OSMF according to histopathological characteristics. According to clinical manifestations, a lesion could be classified into early, moderate, and late stages [33]. Clinical classification was also proposed for OSMF in terms of both clinical staging and functional staging [34]. Scholars have conducted extensive researches on OSMF classifications, but there is no unified classification at present. As what has been stated, the staging and classification of OSMF still need to be further unified and standardized.

### 3.2. Clinical Features

Clinical symptoms of OSMF include ulcer, dry mouth, burning sensation, and mouth opening limitation. As the disease progresses, it may also affect the pharynx and esophagus, leading to fibrosis of the upper digestive tract [30]. In the early stage of OSMF, oral mucosa whitening is an important clinical feature, while mouth opening limitation is caused by fibrosis of the oral mucosa area in the late stage. The typical symptoms of OSMF patients with limited mouth opening directly affect life quality. It should be noticed that many patients with OSMF have anxiety, depression, and stress and show weak social interaction, and the incidence is proportional to the severity of the disease [35].

The clinical diagnostic criteria of OSMF include whitening of the mucous membrane, stiffness, burning sensation, and appearance of characteristic fibrous bands, which are related to the gradual inability to open the mouth. Prodromal symptoms contain a burning sensation in the mouth when eating spicy food, ulcers or recurrent stomatitis, excessive salivation, impaired taste, and occasionally dry mouth with blistering pain [14, 36].

### 3.3. OSMF Histopathological Characteristics

Pathological features include chronic inflammation, excessive collagen deposition in the submucosal connective tissue of the oral mucosa, local inflammation in the lamina propria or deep connective tissue, and degenerative changes in the muscle. The histopathological characteristics of OSMF were first reported by Pindborg and Sirsat; they classified the disease into 4 categories according to the severity of disease progression [33]. Subsequently, Utsunomiya et al. [37] modified the classification system into three stages: early, middle, and late. The early features were mucosal edema changes in the subepithelial region of the mucosa corresponding to the upper lamina propria, accompanied by diffuse infiltration of lymphocytes, but no significant fibrotic changes had occurred. In the middle stage, fibrosis extended to the muscular layer, and transparency began to appear in the hypodermic area. In the late stage, there are extensive areas of fibrosis, with changes from the subepithelial layer to the muscle layer. Collagen fibers show a mixed direction in the early oral submucosal fibrosis, while they show a parallel direction in the late stage, and from early to late, the microvessel density is significantly reduced [38].

### 3.4. Future Diagnosis Method Prospect

The onset of OSMF is recessive; there are cases taking about 2-20 years until symptoms appeared [39]. Early diagnosis method is a problem for this chronic occult disease. At present, the main diagnosis method is tissue biopsy. The global malignant potential of OSMF accounts for 7.6%, and there are some obvious abnormal proliferative changes in the biopsy samples of early OSMF patients [31]. In view of this, it should be emphasized that OSMF can be diagnosed clearly in the early stage to prevent its malignant transformation to OSCC.

Biopsy is still the gold standard for OSMF diagnosis; it is an invasive examination method, and if not handled properly, it may aggravate the pain of OSMF patients. Besides, biopsy often prevents difficulties due to the difficulty of mouth opening. To the best of author's knowledge, liquid biopsies of serum and saliva have been used to extend the methods for detecting oral microenvironment changes [40]. For example, in the detection of saliva lactate dehydrogenase (LDH) levels in patients with oral cancer, oral submucosal fibrosis, and habitual chewing tobacco, it was found that saliva LDH content in precancer and cancer is high, suggesting that LDH may be used as a biomarker to diagnose OSMF possibly [40–42]. In the future, liquid biopsies based on multiple biomarkers are prospected in OSMF diagnosis. Exosomes, as one of the hot areas of liquid biopsy, have rich detectable targets such as protein, DNA, RNA, and lipid. Exosomes have been proven to be a key component in the oral microenvironment [43]. It could be speculated that exosomes have unique advantages and potentials in OSMF screening and prognosis judgment, though the relative applications need further investigations.

It was reported OSMF-associated oral cancer usually presents regional invasive and advanced manifestations, and it is a challenge for clinicians to detect OSMF early malignant changes. Currently, noninvasive diagnostic methods are emerging in the diagnosis of oral precancerous lesions [44]. It can be assumed whether these noninvasive methods can be used for the early diagnosis of OSMF-associated oral cancer, including liquid biopsy, noninvasive optics, cell CT scan, ultrasound elastography, and skin/mucosa CT, and whether they can replace traditional biopsy for OSMF diagnosis in the future. The above supposed applications also need further studies.

## 4. Therapy Strategies

The treatment of OSMF patients varies. Firstly, it is important to stop bad habits such as chewing areca nuts. However, it is difficult to reverse the disease simply by quitting the habit of chewing areca nut in most OSMF patients with moderate to severe disease. Relevant scholars have been committed to using reasonable treatment methods to reverse the progress of the disease.

The most common reason for OSMF patients to see doctors is the difficulty of mouth opening due to oral mucosal rigidity. Therefore, the treatment of OSMF mainly focused on improving mouth opening through medical or surgical methods in the past. Clinical treatment of OSMF patients is divided into nonsurgical or surgical treatment. The latest treatment/management strategies are summarized in Table 1.

### 4.1. Nonsurgical Treatment

#### 4.1.1. Drug Therapy

Nonsurgical treatment includes drug, physical therapy, and nutritional support. Commonly used drugs include steroids, hyaluronidase, chymotrypsin, collagenase, and traditional Chinese medicine. Suppressing the inflammatory response in damaged tissues is considered a reliable strategy to limit the development of fibrosis [45]. Steroids (especially glucocorticoids) and corticosteroids (such as hydrocortisone and dexamethasone) are used to treat OSMF for a long time because of preventing inflammatory response, reducing fibroblast proliferation, and alleviating collagen deposition. Chinese medicine also has a certain effect on the treatment of organ fibrosis. Commonly used Chinese medicine formulas such as salvia/miltiorrhiza have certain effects in OSMF treatment, and it was also reported that combined traditional Chinese and western medicine could get better effects [46].

#### 4.1.2. Physical Therapy

The EZBite opening device is one type of OSMF physical therapy. It was designed for open training which could improve the patient's restricted mouth opening [47].

Hyperbaric oxygen therapy (HBOT) is a commonly used physical therapy in many kinds of disease; it can be used to treat decompression sickness, gangrene gas, and carbon monoxide poisoning. Subsequently, it was first used in the field of dentistry in 1988 to promote periodontal wound healing [48]. Studies have shown that hyperbaric oxygen can promote fibroblast apoptosis and inhibit fibroblast activity by attenuating the production of proinflammatory cytokines (such as IL-1) [49, 50], simultaneously hindering the production of reactive oxygen species [51]. Recently, it was also used for the treatment of OSMF patients.

Photodynamic therapy (PDT) is a medical technology using photodynamic effects to destroy diseased tissues selectively, and its application in oral disease has gradually attracted attention [52]. The advantages of PDT include high targeting, low toxicity, minimal invasion, and repeatable operation system. Nowadays, PDT has been widely used in the treatment of oral cancer [53] and oral precancerous lesions such as oral leukoplakia [54] and lichen planus [55]. However, there are no reports on the efficacy of OSMF at present yet. It still needs to be further studied.

#### 4.1.3. Nutritional Support

Nutritional support is generally recommended for OSMF patients, such as vitamin and iron supplements, a diet rich in minerals, red fruits (such as tomatoes, carrots, and watermelons), green vegetables, and green tea, though there are few studies confirming its direct effectiveness.

Turmeric, with a wide range of therapeutic effects, is a strong antioxidant. It was reported to prevent free radical damage and can alleviate inflammation by reducing histamine while increasing the production of natural cortisone [56]. It has been shown that ginger butter resin, ginger butter, and turmeric extract have therapeutic effects on OSMF [57]. Systemic combined local form has a better therapeutic effect on OSMF rather than the systemic form alone [58]. Lycopene, a red carotenoid, is a powerful antioxidant obtained from tomatoes. Comparing the treatment of lycopene and curcumin in OSMF patients, it was found that lycopene is more effective in improving mouth opening and effective in reducing the burning sensation [59]. In short, it is recommended to eat more natural foods with anti-inflammatory and antioxidant properties, such as honey [60], fruits, and green vegetables, and pay attention to the nutrition balance of OSMF patients.

### 4.2. Surgical Treatment

Surgical treatment has become the first choice for advanced patients. Many surgical methods have been reported in the literature, including cauterization with a knife or laser resection of the fibrous band, and then reconstruction of defects with grafts such as medium thickness skin flap [61], bilateral nasolabial groove flap [62–65], or buccal fat pad [66, 67]. However, the recurrence rate is very high after surgery. Besides, surgical treatment of patients with advanced OSMF can cause a series of complications, such as partial or total necrosis of the flap, hair growth in the mouth, scar outside the mouth, and wound dehiscence [63]. Therefore, surgeons should pay attention to due diligence during the operation to reduce the incidence of complications, and it is necessary to remind patients to give up bad habits, change bad lifestyles, enhance immunity, eat more green vegetables, red fruits, and trace elements, and so on.

### 4.3. Prospects of OSMF Treatment

As summarized in Table 1, various drugs and surgical treatments have been tried to intervene in OSMF disease process, though the effect is limited. Researches and development of new OSMF-targeted drugs or therapies are still necessary.

A new type of therapy has recently been proposed here: stem cell therapy, which is expected to find new breakthroughs for OSMF treatment. Stem cells could promote blood vessel formation by releasing cytokines and growth factors (paracrine effects) and enhancing antioxidant (naturally occurring or foreign) ability to scavenge free radicals [68]. And it may help stimulate the transformation of resident tissue stem cells into new fibroblasts, which may help to remove the biochemical and morphologically altered collagen fibers. The pluripotent stem cells purified in the laboratory have achieved good results in precancerous lesions, OSMF, and periapical cystic lesions. During the treatment of OSMF patients with autologous bone marrow-derived mesenchymal stem cell therapy, the patients have significantly improved mouth opening and the mucosal burning sensation gradually reduced, with no complications such as local calcification or sclerosis at the injection site of stem cells [69]. At present, the sources of stem cells have become abundant. Dental or nonodontogenic mesenchymal stem cells have been effectively used for the regeneration of the maxillofacial region, such as the regeneration of pulp and periodontal ligament; the regeneration of salivary glands, cleft lip, and palate repair; and craniofacial regeneration [70, 71]. Based on the above researches, stem cells are expected to repair oral mucosa and become an effective method for the treatment of OSMF in the future, providing breakthrough points for the progress of OSMF researches.

According to the further research, stem cell-derived exosomes have potential roles in reversing organ fibrosis [72, 73]. To our knowledge, stem cell-derived exosomes have several advantages than stem cells. Mesenchymal stem cell-derived exosomes can be safely used in animal models and show reliable efficacy in heart and liver diseases [74]. It is prospected that the stem cell exosome strategy is the next generation of stem cell technology. Therefore, it can be speculated that the treatment with stem cells and stem cell exosomes in OSMF patients could have anticipated applications.

## 5. Quality of Life of OSMF Patients

The quality of life of OSMF patients decreased significantly (physically and mentally). After ingesting spicy foods, OSMF patients will experience severe mouth burn. The typical symptoms of OSMF patients with limited mouth opening directly affect the level of discomfort in the quality of daily life [35, 91]. Many patients with OSMF have anxiety, depression, and stress and show weak social interaction, and the incidence is proportional to the severity of the disease.

## 6. Prognosis and Malignant Transformation

OSMF is closely related to oral cancer, and it has a high malignant transformation rate of 7%-30% [33]. Studies have also shown that the OSMF-associated OSCC has an earlier age of onset, more aggressive biological characteristics, and a higher rate of metastasis and relapse [92]. It was reported that OSCC caused by OSMF background follows different clinical manifestations, and malignant transformation occurs in younger age groups and has poorer prognosis [93]. The OSMF clinical classifications (concerning opening degree and degree of fibrosis) are not positively associated with the canceration rate due to insufficient evidence. It should be noticed that early OSMF lesions also have a high canceration rate. It is well known that malignant cells are in a very complex microenvironment, and their metabolic pathways have changed, including intermediates involved in oxidative stress, which can enhance metabolic reconnection and promote tumor progression. The role of oxidative stress in PMD such as leukoplakia and OSCC is still under study. Studies have shown that the oxidative stress indicators show a gradually increasing trend from the normal group to the OSMF group and then to the OSCC group [94]. And some studies have proven that compared with the negative control, all antioxidant enzymes are significantly decreased during the leukoplakia progression [95]. These evidences indicate that the relationship between PMD and oxidative stress is well established and that oxidative stress plays an important role in the pathogenesis of oral diseases. Based on these considerations, in the future, reliable and clear diagnostic markers may be found between different free radical molecules or elements in the antioxidant barrier and used as therapeutic targets in clinical practice.

## 7. Conclusion

OSMF is triggered by many etiological factors such as chewing areca nut, high intake of chili, copper toxicity levels in foods and chewing agents, vitamin deficiency and malnutrition, anemia, and genetic susceptibility. Chewing areca nut is still the main cause of OSMF. So far, no specific treatment is available for OSMF, pleading for an urgent need to pursue new treatment methods and to uncover the underlying mechanisms.

**Figure 2:**
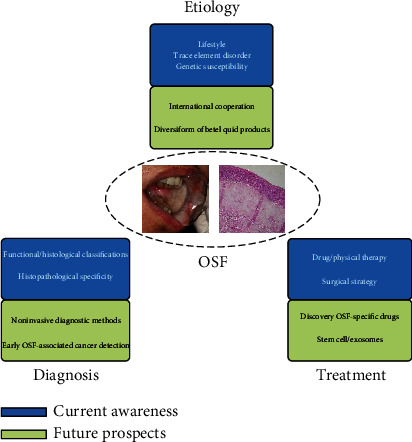
The current awareness and future prospects of OSMF researches.

By summarizing the latest progress of etiology, diagnosis, and treatment of OSMF, prevention first should be advocated. Local health and medical institutions should popularize OSMF knowledge to the public, including not chewing betel nut, paying attention to nutrition intake to reduce the risk of OSMF. Through further understanding of OSMF pathogenesis and related carcinogenesis, with the development of scientific research and medical level, especially in stem cells, exosomes, and other related emerging fields, it is possible to decrease the incidence, recurrence rate, and malignant transformation rate of OSMF (Figure 2).

## Figures and Tables

**Table 1:** The latest treatment/management strategies of OSMF.

Nonsurgical treatment	Medical treatment	Molecular target	References
	Steroids (such as glucocorticoids)	Anti-inflammation	[75–77]
	Corticosteroid (such as hydrocortisone and dexamethasone)	Anti-inflammation	[78–80]
	Hyaluronidase	Hydrolyzed hyaluronic acid, reduced collagen formation	[81–83]
	Chymotrypsin	Hydrolyzed collagen	[84]
	Collagenase	Hydrolyzed collagen	[85]
	Colchicine	Anti-inflammatory neutralizing cytokines (TGF-*β*, IL-4), increased collagen hydrolysis activity	[86]
	Traditional Chinese medicine (such as Salvia miltiorrhiza)	Anti-inflammation, activating blood circulation to dissipate blood stasis	[46]
	Physiotherapy	Molecular target	References
	Hyperbaric oxygen therapy (HBO)	Promote fibroblast apoptosis, inhibit fibroblast activity, and anti-inflammation and antioxidation	[49–51, 87]
	Photodynamic therapy (PDT)	Laser irradiation at a specific wavelength causes an oxidation reaction, resulting in cytotoxicity, cell damage, and death	[53–55]
	EZBite opening device	Improve triceps, improve mouth opening	[47]
	Nutritional support	Molecular target	References
	Ginger butter resin, ginger butter, and turmeric extract	Anti-inflammation; antioxidation; inhibit p53, TGF-*β*, and iNOS; reduce CTGF	[57–59, 88]
	Lycopene	Inhibit fibroblast activity, anti-inflammation, strong antioxidation	[59]
	Honey	Anti-inflammation, antioxidation	[60]
Surgical treatment	Reconstruction of defect area	Advantage	References
	Split-thickness skin graft	Early application	[61]
	Bilateral nasolabial flap	Easy to lift, close to the defect, with minimal swallowing and speech difficulties	[62–65]
	Buccal fat pad	Postoperative complication rate is low and scar formation is low	[66, 67]
	Platysma myocutaneous flap	Less scar formation	[89]
	Superficial temporal fascial flap + skin graft coverage	Postoperative appearance and good mouth opening	[90]
Stem cell therapy	Stem cell source	Molecular target	References
	Bone marrow mesenchymal stem cells, dental pulp stem cells, adipose mesenchymal stem cells, etc.	Release cytokines and growth factors to achieve neovascularization; enhance the ability of antioxidants to scavenge free radicals; remove senescent cells in lesions	[68, 69]
